# Fast sulfate formation from oxidation of SO_2_ by NO_2_ and HONO observed in Beijing haze

**DOI:** 10.1038/s41467-020-16683-x

**Published:** 2020-06-05

**Authors:** Junfeng Wang, Jingyi Li, Jianhuai Ye, Jian Zhao, Yangzhou Wu, Jianlin Hu, Dantong Liu, Dongyang Nie, Fuzhen Shen, Xiangpeng Huang, Dan Dan Huang, Dongsheng Ji, Xu Sun, Weiqi Xu, Jianping Guo, Shaojie Song, Yiming Qin, Pengfei Liu, Jay R. Turner, Hyun Chul Lee, Sungwoo Hwang, Hong Liao, Scot T. Martin, Qi Zhang, Mindong Chen, Yele Sun, Xinlei Ge, Daniel J. Jacob

**Affiliations:** 1 Jiangsu Key Laboratory of Atmospheric Environment Monitoring and Pollution Control, School of Environmental Science and Engineering, Nanjing University of Information Science and Technology, Nanjing, 210044 China; 2 John A. Paulson School of Engineering and Applied Sciences, Harvard University, Cambridge, MA 02138 USA; 3 State Key Laboratory of Atmospheric Boundary Layer Physics and Atmospheric Chemistry, Institute of Atmospheric Physics, Chinese Academy of Sciences, Beijing, 100029 China; 4 Department of Atmospheric Sciences, School of Earth Sciences, Zhejiang University, Hangzhou, 310027 China; 5 School of Atmospheric Sciences, Nanjing University, Nanjing, 210023 China; 6 State Environmental Protection Key Laboratory of Formation and Prevention of Urban Air Pollution Complex, Shanghai Academy of Environmental Sciences, Shanghai, 200233 China; 7 State Key Laboratory of Urban and Regional Ecology Research Center for Eco-Environmental Sciences, Chinese Academy of Sciences, Beijing, 100085 China; 8 State Key Laboratory of Severe Weather, Chinese Academy of Meteorological Sciences, Beijing, 100081 China; 9 Department of Energy, Environmental and Chemical Engineering, Washington University in Saint Louis, St. Louis, MO 63130 USA; 10 Samsung Advanced Institute of Technology, Suwon-si, Gyeonggi-do 16678 Republic of Korea; 11 Department of Environmental Toxicology, University of California Davis, Davis, CA 95616 USA

**Keywords:** Atmospheric chemistry, Atmospheric chemistry, Environmental monitoring

## Abstract

Severe events of wintertime particulate air pollution in Beijing (winter haze) are associated with high relative humidity (RH) and fast production of particulate sulfate from the oxidation of sulfur dioxide (SO_2_) emitted by coal combustion. There has been considerable debate regarding the mechanism for SO_2_ oxidation. Here we show evidence from field observations of a haze event that rapid oxidation of SO_2_ by nitrogen dioxide (NO_2_) and nitrous acid (HONO) takes place, the latter producing nitrous oxide (N_2_O). Sulfate shifts to larger particle sizes during the event, indicative of fog/cloud processing. Fog and cloud readily form under winter haze conditions, leading to high liquid water contents with high pH (>5.5) from elevated ammonia. Such conditions enable fast aqueous-phase oxidation of SO_2_ by NO_2_, producing HONO which can in turn oxidize SO_2_ to yield N_2_O.This mechanism could provide an explanation for sulfate formation under some winter haze conditions.

## Abstract (web summary)

How sulfur dioxide emitted through coal combustion is oxidized to sulfate particles during winter haze pollution events has been the subject of debate. Here, the authors show that rapid oxidation takes place by nitrogen dioxide and nitrous acid, producing nitrous oxide together with sulfate.

## Introduction

Beijing experiences severe air pollution events in winter, commonly called winter haze. The concentration of fine particulate matter with aerodynamic diameter less than or equal to 2.5 μm (PM_2.5_) can exceed 200 μg m^−3^ on a 24-h average basis during these events^1^, considerably higher than the 24-h Chinese National Ambient Air Quality Standard of 75 μg m^−3^. Winter haze events are often associated with high relative humidity (RH)^2–5^ and a major contribution of sulfate to total PM_2.5_^6^. Sulfate is produced in the atmosphere by oxidation of sulfur dioxide (SO_2_) emitted from coal combustion^7,8^. But the photochemical oxidants known to drive atmospheric oxidation of SO_2_ (hydroxyl radical, hydrogen peroxide, ozone) have very low concentrations under typical winter haze conditions^7,9,10^. This has led to considerable debate regarding the mechanisms responsible for sulfate formation in winter haze^9,11–13^.

The high-RH conditions characteristic of winter haze cause particulate matter to take up water, enabling aqueous-phase pathways for SO_2_ oxidation. SO_2_ is a weak acid with moderate water solubility (Henry’s law constant *K*_*H*_ = 1.2 M atm^−1^ at 298 K) that dissociates in water to form bisulfite (HSO_3_^−^; *pK*_*a,1*_ = 1.9 at 298 K) and sulfite (SO_3_^2−^; *pK*_*a,2*_ = 7.2 at 298 K). Bisulfite and sulfite are converted to sulfate by a number of aqueous-phase oxidants with rates dependent on pH^14^. As the air cools at night or through rising motions the haze can turn to fog and low clouds (RH > 100%), increasing the atmospheric liquid water content (LWC) by orders of magnitude and hence the importance of SO_2_ aqueous-phase oxidation pathways.

Most previous studies of sulfate formation during Beijing haze events have focused on mechanisms taking place in the ubiquitous haze particles (RH < 100%) rather than in the more sporadic fog and cloud (RH > 100%)^13,15–17^. Haze particles are concentrated aqueous solutions with pH that can be estimated from standard thermodynamics^18^. Aqueous-phase oxidation of SO_2_ by nitrogen dioxide (NO_2_) in haze has been proposed^9,11,13^, with NO_2_ originating from vehicular emissions, but requires higher pH than the 4–5 range inferred from thermodynamic calculations^19–21^. Some studies have suggested that oxidation by NO_2_ would be enhanced by fog^9,13,17^. Aqueous-phase autoxidation of SO_2_ by molecular oxygen catalyzed by transition metal ions (TMI) has been proposed^22,23^ but is poorly constrained due to the lack of information on TMI concentration, complexation, and solubility^24^. A recent study suggests that aqueous-phase oxidation by hydrogen peroxide (H_2_O_2_) in haze could be significant^10^. Yet another suggestion is that some of the reported sulfate could actually be hydroxymethanesulfonate (HMS) produced by in-cloud complexation of HSO_3_^−^ and SO_3_^2−^ with formaldehyde (HCHO)^25,26^.

Here we present detailed chemical observations during a Beijing haze event in December 2016 where PM_2.5_ concentrations reached 400 μg m^−3^. We observe fast sulfate production as RH increases over the course of the event, leading to extensive nighttime fog and low clouds, and find a concurrent increase of nitrous oxide (N_2_O). N_2_O is a product of aqueous-phase SO_2_ oxidation by dissolved nitrous acid (HONO)^27–29^, and observations of HONO during the event support this sulfate formation mechanism. Most of the HONO appears in turn to be produced by aqueous-phase SO_2_ oxidation by NO_2_, leading us to propose a two-step fog-enabled mechanism for sulfate formation during winter haze events.

## Results

### Field observations

**Fig. 1:**
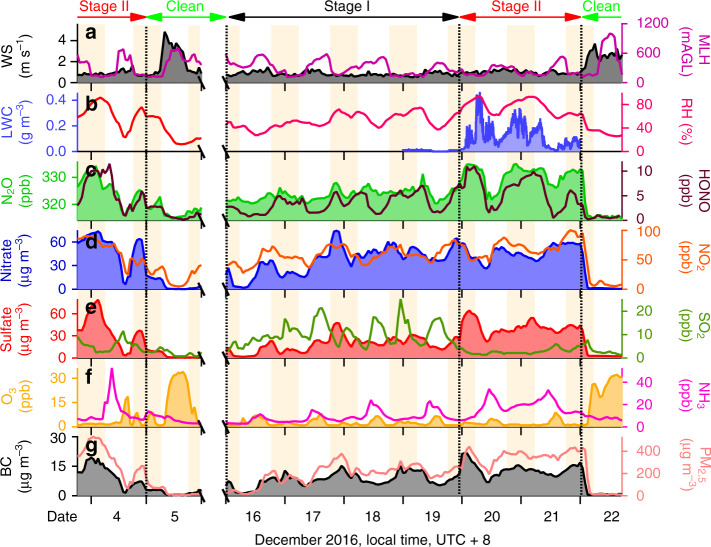
Chronology of a winter haze event. The figure shows the time series of meteorological parameters and chemical species measured in Beijing on December 4–22, 2016. The measurements were made from the roof of an Institute of Atmospheric Physics (IAP) building except for liquid water content (LWC) measured at the Beijing Observatory of the China Meteorological Administration 20 km to the southeast, and PM_2.5_ concentration measured at the Beijing Olympic Center Observatory 4 km to the northeast. UTC+8 denotes Coordinated Universal Time + 8 h and represents local solar time in hours. **a** 10-m wind speed (WS) and mixing layer height (MLH) above ground level (AGL). **b** LWC and relative humidity (RH); **c** N_2_O and HONO concentrations; **d** PM_1_ nitrate and NO_2_ concentrations; **e** PM_1_ sulfate and SO_2_ concentrations; **f** ozone (O_3_) and ammonia (NH_3_) concentrations; and **g** black carbon (BC) and PM_2.5_ concentrations. The campaign sampled the tail end of a haze event on December 4, terminated by passage of a cold front on December 5 and followed by variable conditions on December 6–15 (not shown, note break in time axis). It then sampled an extended pollution episode on December 16–22 with initially moderate RH of 40–75% (Stage I), followed by high RH (>75%) including dense nighttime fog at the Beijing Observatory (Stage II), and ending on December 22 with the passage of a cold front. Nighttime periods are shaded. Sulfate and nitrate measurements are from the HR-AMS instrument with a size cut of 1-μm diameter (see text).

Figure 1 shows the time series of selected variables measured at our field site on the rooftop of an Institute of Atmospheric Physics (IAP) building in urban Beijing during December 4–22, 2016. The start of the campaign on December 4 sampled the end of a haze event that terminated on December 5 with passage of a cold front. Variable conditions were observed during December 6–15 (data not shown). An extended haze event then developed over the December 16–22 period, with 24-h average PM_2.5_ exceeding 200 μg m^−3^ for 6 successive days before a cold front swept in with clean air on December 22.

Wind speed during the December 16–22 haze event was persistently low in the range of 0.3–1.5 m s^−1^ and the mixed layer height (MLH) was less than 600 m above ground level (AGL), decreasing to 300 m at night. The early part of the event on December 16–19 (labeled Stage I in Fig. 1) had moderate RH in the 40–75% range. On December 20–21 (Stage II) the RH rose to above 75% as temperatures cooled to an average of 271 K at night, and dense nighttime fog with LWC as high as 0.5 g m^−3^ was observed at the Beijing Observatory meteorological station 20 km to the south (Fig. 1). Beijing International Airport also reported fog during that period (Supplementary Fig. 1). Dense fog was not observed at our site, but the visibility dropped below a few hundred meters and low clouds formed just 50 m above ground (Supplementary Fig. 2).

PM_2.5_ concentrations rose to over 400 μg m^−3^ during the high-RH period (Stage II) in concert with a rise in sulfate, while nitrate remained at the same concentration as in Stage I (47 μg m^−3^). Black carbon (BC) increased from 9.4 to 13.1 μg m^−3^. SO_2_ concentrations were relatively high in Stage I but nearly depleted in Stage II, indicating rapid oxidation of emitted SO_2_ to sulfate.

**Fig. 2:**
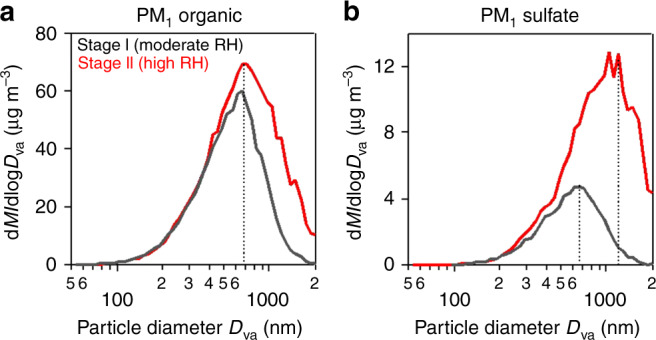
Size distributions of organic and sulfate particles. The Figure shows mass-based size distributions of (**a**) organic and (**b**) sulfate particles in Stages I and II of the December 16–22, 2016 haze event. *M* denotes mass and *D*_va_ denotes particle vacuum aerodynamic diameter. The measurements were made by the HR-AMS instrument with 50% size cut at 1-μm diameter, hence the data are shown as PM_1_ (particulate matter with less than 1-μm diameter). Mass modal diameters are shown as dotted lines.

Figure 2 shows that the sulfate particles measured by high-resolution aerosol mass spectrometer (HR-AMS) shifted to larger sizes during Stage II while the organic particles did not, consistent with sulfate formation taking place in fog and low cloud (cloud-mediated coagulation would have affected both sulfate and organic particles). Mean PM_2.5_ increased from 210 μg m^−3^ in Stage I to 330 μg m^−3^ in Stage II, while sulfate measured by HR-AMS increased fourfold from 10 to 40 μg m^−3^. The sampling efficiency of the HR-AMS instrument (PM_1_) drops off rapidly for particles above 1-μm diameter^30^, implying that actual sulfate levels during Stage II were probably much higher than measured. Indeed, PM_2.5_ sulfate concentrations measured at the site by on-line ion chromatography (URG-9000D Ambient Ion Monitor) were 1.5-2 times larger than the HR-AMS measurements during Stage II (Supplementary Fig. 3). Some of the sulfate particles could even be larger than PM_2.5_ due to swelling at high RH.

### Evidence for SO_2_ oxidation by HONO

A remarkable feature of the observations in Stage II is the large rise in N_2_O concentrations concurrently with sulfate. N_2_O is a major greenhouse gas with a globally dominant biogenic source^31^. It is chemically inert in the troposphere. Vehicles and coal combustion may be a significant source of N_2_O in Beijing^32^, but this would not explain the N_2_O rise in Stage II because no parallel rise was observed for BC (Fig. 1).

**Fig. 3:**
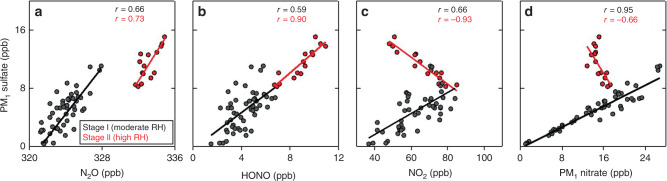
Relationships between sulfate and nitrogen oxide concentrations. Data are shown for Stages I and II of the December 16–22, 2016 haze event. Concentrations of sulfate and nitrogen oxides are expressed in common parts per billion (ppb) units (1 μg m^−3^ sulfate = 0.23 ppb at *T* = 273.15 K). Individual points are hourly mean values from Fig. 1 during nighttime hours (19:00–6:00 local time). The figure shows scatterplots of PM_1_ sulfate versus **a** nitrous oxide (N_2_O); **b** nitrous acid (HONO); **c** nitrogen dioxide (NO_2_); **d** PM_1_ nitrate. Pearson correlation coefficients (*r*) and reduced-major-axis regression lines are shown separately for Stages I and II.

Figure 3 shows the relationships between the concentrations of sulfate and different nitrogen oxide species (N_2_O, HONO, NO_2_, and PM_1_ nitrate) observed in Stages I and II. The relationships are shown only for nighttime hours (19:00–06:00) to minimize strong common dependences on diurnal changes in mixed layer depth, and to avoid the effect of fast HONO photolysis in the daytime. Sulfate correlates positively with all species in Stage I, which may reflect common dependences on atmospheric mixing and ventilation. In Stage II, sulfate is positively correlated with N_2_O (including a step increase) and with HONO, but negatively correlated with NO_2_ and nitrate. This suggests a change in the regime for sulfate production in Stage II with associated production of N_2_O. Looking back at the tail end of the previous haze event on December 4, which also featured high-RH conditions, we again see elevated N_2_O together with sulfate (Fig. 1).

N_2_O is a product of the aqueous-phase oxidation of SO_2_ by HONO^28,29^. HONO is moderately soluble in water (Henry’s law constant *K*_*H*_ = 49 M atm^−1^ at 298 K) and dissociates as a weak acid (*pK*_*a*_ = 3.2 at 298 K) to increase its partitioning in the aqueous phase^33^. The aqueous-phase oxidation of SO_2_ by HONO can be expressed stoichiometrically as follows^28,29^:

R1 $${\mathrm{2N(III)}} + {\mathrm{2S(IV)}} \to {\mathrm{N}}_2{\mathrm{O}} \uparrow + {\mathrm{2S(VI)}} + {\mathrm{other}}\,{\mathrm{products}}.$$

Here N(III) ≡ HONO(aq) + NO_2_^−^ denotes the dissolved HONO species, S(IV) ≡ SO_2_∙H_2_O + HSO_3_^−^ + SO_3_^2−^ denotes the dissolved SO_2_ species, and S(VI) ≡ H_2_SO_4_(aq) + HSO_4_^−^ + SO_4_^2−^ denotes the sulfate species. The other products may include H_2_O or H^+^ depending on the speciation of N(III), S(IV), and S(VI). A laboratory study by Martin et al.^28^ gives a rate expression for sulfate formation from reaction (R1) at pH < 4:

1 $$d\left[ {{\mathrm{S}}\left( {{\mathrm{VI}}} \right)} \right]/dt = k_1\left[ {{\mathrm{H}}^ + } \right]^{0.5}\left[ {{\mathrm{N}}\left( {{\mathrm{III}}} \right)} \right]{\mathrm{[S(IV)]}},$$

where *k*_1_ = 142 M^−3/2^ s^−1^. Another study by Oblath et al.^27^ gives a rate expression

2 $$d\left[ {{\mathrm{S}}\left( {{\mathrm{VI}}} \right)} \right]/dt = k_1^\prime \left[ {{\mathrm{H}}^{\mathrm{ + }}} \right]\left[ {{\mathrm{N}}\left( {{\mathrm{III}}} \right)} \right]{\mathrm{[S}}\left( {{\mathrm{IV}}} \right],$$

with *k'*_1_ = 4800 M^−2^ s^−1^ for 3 < pH < 7. Even though these rate expressions show positive [H^+^] dependences, the rates actually increase with pH because both [S(IV)] and [N(III)] are inversely dependent on [H^+^] over the relevant pH range.

The increase of N_2_O in Stage II concomitant with sulfate suggests that reaction (R1) could be a source of sulfate. However, the slopes of the sulfate-N_2_O regression lines in Fig. 3 are similar for Stages I and II and not consistent with the 2:1 stoichiometry of reaction (R1). A possible explanation is that the PM_1_ sulfate measurements underestimated total sulfate concentrations during Stage II, as shown above. In addition, it is likely that the correlations and slopes are mainly driven by mixing rather than chemistry, as is frequently observed in polluted air masses^34,35^. The signature of the reaction (R1) taking place in Stage II would then be manifested by the step increase in N_2_O between the two Stages.

### Importance of fog and cloud

Reaction (R1) requires fog or cloud to proceed at an appreciable rate. LWCs in haze are too low. It also requires a relatively high pH. The mean gaseous ammonia concentration observed during Stage II was 14 ppb (Fig. 1), typical of previous observations during haze events^36^ and mainly attributable to emissions from fuel combustion^37^. Fog has a much higher pH than haze under high-ammonia conditions because of efficient scavenging of ammonia at high LWC. Whereas ammonia volatility limits the pH of haze aqueous solutions to a 4–5 range even with ammonia in large excess^19,20,38–40^, the corresponding pH range in fog is 6–7^9,13,21,41,42^. Higher pH in haze can be achieved if dust is a significant component^9,21,42^ but low LWC is still a limitation. PM_2.5_ concentrations of dust cations (Ca^2+^, Mg^2+^) were low at our site, as described in the “Methods” section.

Our sampling site did not actually experience fog during Stage II, but the cloud deck extended down to 50 m (Supplementary Fig. 2), and surface air would have been processed by that low cloud and/or by fog elsewhere. We estimate a fog/cloud pH of 5.7 on the basis of our mean measured value of 14 ppb total ammonia to be partitioned into the fogwater; a fog LWC of 0.15 g m^−3^; sulfate, nitrate, and chloride PM_2.5_ present as their ammonium salts; and a temperature of 271 K (see “Methods”). Past observations for Beijing in winter indicate a fog/cloud pH range of 4.7–6.9^7,11,26,43,44^. Sensitivity to pH will be examined in the “Discussion” section.

We can estimate the e-folding lifetime for SO_2_ oxidation by HONO in nighttime fog on the basis of a fog with pH 5.7 and LWC of 0.15 g m^−3^, and assuming a mean nighttime total HONO concentration of 9 ppb as measured during Stage II (Fig. 1). This involves applying the rate expression for reaction (R1) with Henry’s law and acid dissociation constants computed at 271 K (Supplementary Table 1). We find a fogwater nitrite (N(III), mainly as NO_2_^−^) concentration of 2.2 μmol L^−1^, which leads to an e-folding SO_2_ lifetime of 3.8 h using the rate expression of Martin et al.^28^ extended to pH 5.7 but 79 h using the rate expression of Oblath et al.^27^. The former would imply a major role of HONO as SO_2_ oxidant while the latter would imply an insignificant role. As we will see, the HONO concentration in fog could actually be much higher than measured at our site, which would increase the importance of reaction (R1). The observed increase of N_2_O in Stage II does suggest an important role for reaction (R1).

### Evidence for SO_2_ oxidation by NO_2_ and production of HONO

A remarkable result in Fig. 3 is the positive correlation of sulfate with HONO during Stage II, and the negative correlations with NO_2_ and nitrate. Aqueous-phase loss of NO_2_ during haze and fog is generally thought to be driven by particle-phase disproportionation to HONO and HNO_3_^11^, but if this were the case we would expect an increase in nitrate during Stage II in contrast to what was observed (Figs. 1 and 3). Aqueous-phase oxidation of S(IV) by NO_2_ (aq) in fog is an alternative explanation for the depletion of NO_2_ and produces both HONO and sulfate^28^, which would be consistent with the positive correlation observed between the two (Fig. 3):

R2 $${\mathrm{S}}\left( {{\mathrm{IV}}} \right) + {\mathrm{2NO}}_2\left( {{\mathrm{aq}}} \right) + {\mathrm{H}}_2{\mathrm{O}} \to {\mathrm{S}}\left( {{\mathrm{VI}}} \right) + {\mathrm{2H}}^ + + {\mathrm{2NO}}_2^ -.$$

Laboratory studies give a rate expression for reaction (R2) as

3 $$d\left[ {{\mathrm{S}}\left( {{\mathrm{VI}}} \right)} \right]/dt = k_2[{\mathrm{NO}}_{\mathrm{2}}\left( {{\mathrm{aq}}} \right)][{\mathrm{S}}({\mathrm{IV}})],$$

with *k*_2_ = 2 × 10^6^ M^−1^ s^−1^ for the pH range 5.8–6.4 (Lee and Schwartz^45^) and *k*_2_ = 1.2–1.5 × 10^7^ M^−1^ s^−1^ for the pH range 5.3–6.8 (Clifton et al.^46^). For a mean nighttime NO_2_ concentration of 50 ppb during Stage II (Fig. 1), and a fog with LWC = 0.15 g m^−3^ and pH = 5.7, we find an SO_2_ e-folding lifetime of 1–7 min against loss by reaction (R2) depending on which value of *k*_2_ is used, sufficiently short in any case for SO_2_ depletion. Reaction (R2) further produces N(III) as ${\mathrm{NO}}_2^ -$, which in a fog of pH 5.7 would remain in the aqueous phase and may thus go on to oxidize SO_2_ by reaction (R1). If reaction (R1) is sufficiently fast, following the rate expression of Martin et al.^28^, then a steady state would be established at night between production of ${{\mathrm{NO}}_2^ -}$ in the fog by reaction (R2) and loss by reaction (R1), resulting in an effective sulfate mass yield of 2 from reaction (R2).

An SO_2_ oxidation mechanism in nighttime fog involving reaction (R2) followed by reaction (R1) would be consistent with our observed enhancement of N_2_O. In that mechanism, one mole of N_2_O is produced for every three moles of SO_2_ oxidized. Starting from a SO_2_ level of 20 ppb in Stage I (Fig. 1), complete oxidation of that SO_2_ to sulfate would produce 7  ppb N_2_O, consistent with the ≈5 ppb increase of N_2_O observed between Stage I and Stage II (Fig. 3). The mechanism both produces and consumes HONO in the oxidation of SO_2_, whereas N_2_O is a terminal product, which may explain why HONO shows a positive correlation with sulfate in Stage II but not a step increase. One would similarly expect one mole of NO_2_ to be consumed for every 0.5–1.5 mole of SO_2_ oxidized, depending on whether oxidation by (R2) is followed by (R1). The sulfate-NO_2_ slope in Fig. 3 is only −0.2 mol mol^−1^, which could suggest additional NO_2_ sinks associated with fog, an underestimate of sulfate in the Stage II observations as previously discussed, or a dominance of atmospheric mixing in determining the slope.

## Discussion

**Fig. 4:**
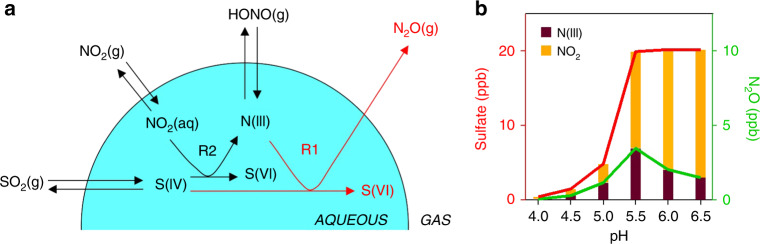
SO_2_ oxidation mechanism. **a** Illustration of the mechanism for SO_2_ oxidation to sulfate by NO_2_ and HONO in fog and cloud. Here S(IV) ≡ SO_2_∙H20 + HSO_3_^−^ +  SO_3_^2−^ denotes the different forms of dissolved SO_2_, S(VI) ≡ H_2_SO_4_(aq) + HSO_4_^−^ + SO_4_^2−^ denotes the different forms of sulfate, and N(III) ≡ HNO_2_(aq) + NO_2_^−^ denotes the different forms of dissolved HONO. S(IV) is oxidized in the aqueous phase by dissolved NO_2_ (reaction (R2)), producing N(III) which may oxidize additional S(IV) and produce N_2_O by reaction (R1). **b** pH-dependent sulfate and N_2_O production from the (R2) + (R1) mechanism. The plot shows accumulated concentrations of sulfate and N_2_O after 5 h in a nighttime fog air parcel simulation initialized with [SO_2_] = 20 ppb, [NO_2_] = 80 ppb, and [HONO] = 5 ppb, with a fog LWC = 0.15 g m^−3^ and *T* = 271 K. Contributions from aqueous-phase SO_2_ oxidation by HONO (reaction (R1)) and NO_2_ (reaction (R2)) are shown separately. A sulfate production of 20 ppb implies complete oxidation of SO_2_ over the 5 h.

Figure 4a illustrates our proposed mechanism for sulfate formation involving reactions (R1) and (R2) in nighttime fog and cloud associated with winter haze events. We conducted air parcel model calculations to study the pH dependence of sulfate formation in this mechanism. For reaction (R1) we used the rate expression from Martin et al.^28^, because the much slower rate expression of Oblath et al.^27^ would not explain the observed N_2_O enhancement. For reaction (R2) we followed the rate constant (*k*_2_) estimates of Lee and Schwartz^45^ as 1.4 × 10^5^ M^−1^ s^−1^ for pH < 5 and 2 × 10^6^ M^−1^ s^−1^ for pH > 6, with linear interpolation between these two pH ranges. Henry’s law and acid dissociation equilibrium constants for SO_2_, NO_2_, and HONO are in Supplementary Table 1. The air parcel was initialized with concentrations taken from the field observations during Stage I including [SO_2_] = 20 ppb, [NO_2_] = 80 ppb, [HONO] = 5 ppb, and then allowed to evolve as a closed system for 5 h in a nighttime fog with LWC = 0.15 g m^−3^ and *T* = 271 K. The time scale for equilibration between the gas and aqueous phases in fog is less than a few minutes^47^, so that Henry’s law can be applied to all three gases. In the case of HONO, N(III) has a lifetime against oxidation of S(IV) of 1.5 h for 20 ppb SO_2_ and pH = 5.7, and this lifetime becomes longer as SO_2_ is depleted.

As shown in Fig. 4b, we find in this air parcel model that reactions (R1) and (R2) are sufficiently fast for complete conversion of SO_2_ to sulfate at pH > 5.5, with a maximum contribution from reaction (R1) at pH 5.5. At higher pH, the faster kinetics of reaction (R2) decrease the role of reaction (R1) in competing for SO_2_ oxidation, resulting in a lower yield of N_2_O. The N_2_O yield would also be low (<2 ppb) if we used the faster kinetics for (R2) from Clifton et al.^46^. The observed N_2_O enhancement of ≈5 ppb is most consistent with the kinetics of Lee and Schwartz^45^ for (R2) and Martin et al.^28^ for (R1), with a fog/cloud pH of 5.5 (Fig. 4b), but uncertainties are obviously large. Further analysis will require better kinetic information for reactions (R1) and (R2). Decreasing ammonia emissions to bring cloud pH below 5 would shut down the mechanism (Fig. 4b), but other SO_2_ oxidation pathways may then take over such as TMI-catalyzed autoxidation^22^.

The role of (R2) + (R1) as a source of HONO and N_2_O is of interest, considering that HONO photolysis is a major source of radicals during winter haze^48^ and that N_2_O is a major anthropogenic greenhouse gas. Previous studies have found that HONO in Beijing haze has a large source from direct vehicular emissions^48,49^ and this could explain the rise of HONO observed during Stage I (Fig. 1). However, the doubling of HONO concentrations from Stage I to Stage II suggests that (R2) could be an important source of HONO during haze events. With regard to N_2_O, the most relevant comparison is to the national anthropogenic source for China, estimated to be 2141 Gg a^−1^ with a dominant contribution from agriculture^50^. For a rough estimate, let us assume that (R2) + (R1) is the dominant SO_2_ sink during high-RH winter haze, accounting for ~8% of winter days (data downloaded from https://rp5.ru/), and that the N_2_O molar yield is 20% based on the upper limit (pH 5.5) of Fig. 4b. The Multi-resolution Emission Inventory for China estimates a national SO_2_ emission of 13.4 Tg a^−1^ in 2016^51^, which would then imply a corresponding N_2_O source of 36.8 Gg a^−1^. This is small compared with the national inventory total, but not negligible as a component of N_2_O emission from the energy sector estimated to be 232.7 Gg a^−1^ in 2012^50^.

In summary, we have shown from field observations of an extended winter haze PM_2.5_ pollution event in Beijing that aqueous-phase oxidation of SO_2_ by NO_2_ and HONO in nighttime fog and low cloud provides a plausible mechanism for explaining the rapid production of sulfate PM_2.5_. Production of sulfate in fog and cloud is consistent with the observed shift in the sulfate size distribution to larger sizes. High-RH conditions with widespread fog and low cloud formation are typical of severe winter haze events in Beijing^2,26^. This provides high LWCs for aqueous-phase reactions to occur, together with high pH (>5.5) from efficient uptake of ammonia. Based on available aqueous-phase kinetic data, such high-LWC high-pH conditions should allow fast oxidation of SO_2_ by NO_2_ to produce HONO, and subsequent fast oxidation of SO_2_ by HONO to produce N_2_O. There remains large uncertainty in these kinetic data. But such a mechanism is consistent with our field observations of N_2_O enhancement, HONO enhancement, NO_2_ depletion, and near-complete SO_2_ depletion concurrent with fast sulfate production as RH increased during the haze event. Further work should target better understanding of the laboratory kinetics and products of the aqueous-phase reactions of SO_2_ with NO_2_ and HONO.

## Methods

### Field campaign

The field campaign was conducted at the Tower Site of the IAP, Chinese Academy of Science (39°58′N, 116°22′E) in Beijing, China from 4 to 22 December of 2016. This site is located around the 3rd ring road of north Beijing, surrounded by residential infrastructure and an arterial road to the east (360 m). Measurements were made from a rooftop laboratory 8 m above ground and with no interference from neighboring buildings. All data presented in this paper were hourly averaged (local time, UTC+8).

A HR-AMS was deployed during the field campaign to obtain chemical composition and size distributions of non-refractory particulate matter smaller than 1-μm diameter (NR-PM_1_). A shared PM_2.5_ cyclone inlet (Model URG-2000-30ED) and a diffusion dryer were used prior to the sampling. Detailed information on the operation of HR-AMS during the sampling campaign can be found in previous literature^4,52^. Additional measurements of aerosol composition were made with a URG-9000D Ambient Ion Monitor for water-soluble ions including a BGI-VSCC PM_2.5_ cyclone upstream. Anion analysis was performed using the IonPac AS19 hydroxide-selective anion-exchange column, which can effectively separate sulfate from HMS.

PM_2.5_ mass concentration was measured by a TDMS-TEOM PM_2.5_ analyzer (Thermo Fisher Scientific, Model 1405) at the Beijing Olympic Center Observatory, which is 4 km to the northeast of the sampling site. Fog LWC was measured by a TP/W VP-3000 ground-based 12-channel microwave radiometer (Radiometrics Corp.) at the Beijing Observatory of the China Meteorological Administration (CMA), 20 km to the south of our sampling site.

Gaseous and meteorological data were also collected at the site. An Aerodyne high-resolution time-of-flight chemical ionization mass spectrometer measured HONO concentrations^53^. N_2_O concentration was measured with a real-time CH_4_/N_2_O Analyzer (Los Gatos Research, Inc.). Concentrations of O_3_, SO_2_ (precision 0.5 ppb), and NO_2_ were measured with Thermo Fisher Scientific instruments (Models 49*i*, 43*i*, 42*C*), and NH_3_ by a Los Gatos Research analyzer. Vertical profiles of meteorological parameters, including wind speed and direction, temperature, and RH were measured from the IAP 325-m meteorological tower.

Figure 1 shows the time series of fog LWC measured at the Beijing CMA Observatory, 20 km to the south of our IAP sampling site. No fog was observed at the Observatory during Stage I (December 16–19) but pervasive nighttime fog was observed during Stage II (December 20–21) with a mean LWC of 0.15 g m^−3^ and maximum of 0.4 g m^−3^. CMA forecasts advised for strong fog (visibility 50–200 m) and extra-strong fog (visibility <50 m) across the North China Plain during the Stage II period (http://products.weather.com.cn/product/Index/index/procode/YB_W_24.shtml).

Previous studies have reported fog LWCs of 0.2–0.3 g m^−3^ in the North China Plain in association with winter haze events^54,55^.

### Calculation of fog/cloud pH

We estimated fog/cloud pH values during Stage II by assuming a pre-fog atmosphere with the mean composition observed at the IAP field site, and adding to that atmosphere an LWC of 0.15 g m^−3^. The IAP field site did not experience fog during Stage II, but cooling of a few degrees would have caused fog to form (as apparent in the low clouds observed 50 m above the site, Supplementary Fig. 2) and drive partitioning of gases into the aqueous phase. We can then estimate the fog/cloud pH from the partitioning of the relevant chemicals initially present in pre-fog air as defined by the mean conditions of Stage II (Fig. 1). This includes 14 ppb NH_3,_ 2 ppb SO_2_, and PM_2.5_ with electroneutral composition [SO_4_^2−^] = 3 × 10^−3^ mol L^−1^, [NO_3_^−^] = 6 × 10^−3^ mol L^−1^, [Cl^−^] = 1 × 10^−3^ mol L^−1^, and [NH_4_^+^] = 1.3 × 10^−2^ mol L^−1^. We also include 400 ppm CO_2_, and neglect organic acids which are low under winter haze conditions^56^. Alkaline dust would increase the pH and is found to be important in precipitation data for winter Beijing^14,57^ but our mean Stage II PM_2.5_ measurements show [Ca^2+^] = 1.2 × 10^−5^ mol L^−1^ and [Mg^2+^] = 2.1 × 10^−5^ mol L^−1^ for the principal crustal cations, negligible relative to [NH_4_^+^]. Thus we ignore the contribution of dust in the pH calculation, acknowledging that this may cause an underestimate of pH since dust could be present in larger particle sizes. We performed the pH calculation for a temperature of 271 K with the Henry’s law and acid dissociation constants in Supplementary Table 1. We obtain in this manner a fog/cloud pH of 5.7.

## Supplementary information

Supplementary Information

## Data Availability

Datasets including time series of species concentrations and meteorological variables during the campaign are available at 10.7910/DVN/FS7746.

## References

[1] Sun J (2019). Investigating the PM_2.5_ mass concentration growth processes during 2013–2016 in Beijing and Shanghai. Chemosphere.

[2] Pendergrass DC, Shen L, Jacob DJ, Mickley LJ (2019). Predicting the impact of climate change on severe wintertime particulate pollution events in Beijing using extreme value theory. Geophys. Res. Lett..

[3] Shen L, Jacob DJ, Mickley LJ, Wang Y, Zhang Q (2018). Insignificant effect of climate change on winter haze pollution in Beijing. Atmos. Chem. Phys..

[4] Zhao J (2019). Organic aerosol processing during winter severe haze episodes in Beijing. J. Geophys. Res. Atmos..

[5] Huang RJ (2020). Contrasting sources and processes of particulate species in haze days with low and high relative humidity in winter time Beijing. Atmos. Chem. Phys. Discuss..

[6] Liu Y (2019). High-time-resolution source apportionment of PM_2.5_ in Beijing with multiple models. Atmos. Chem. Phys..

[7] Xue J (2016). Sulfate formation enhanced by a cocktail of high NO_x_, SO_2_, particulate matter, and droplet pH during haze-fog events in megacities in China: an observation-based modeling investigation. Environ. Sci. Technol..

[8] Tian J (2018). Primary PM_2.5_ and trace gas emissions from residential coal combustion: assessing semi-coke briquette for emission reduction in the Beijing-Tianjin-Hebei region, China. Atmos. Environ..

[9] Cheng Y (2016). Reactive nitrogen chemistry in aerosol water as a source of sulfate during haze events in China. Sci. Adv..

[10] Liu T, Clegg SL, Abbatt JPD (2020). Fast oxidation of sulfur dioxide by hydrogen peroxide in deliquesced aerosol particles. Proc. Natl Acad. Sci. USA..

[11] Wang G (2016). Persistent sulfate formation from London Fog to Chinese haze. Proc. Natl Acad. Sci. USA..

[12] Gen M, Zhang R, Huang DD, Li Y, Chan CK (2019). Heterogeneous SO_2_ oxidation in sulfate formation by photolysis of particulate nitrate. Environ. Sci. Technol. Lett..

[13] Xue J (2019). Efficient control of atmospheric sulfate production based on three formation regimes. Nat. Geosci..

[14] Seinfeld, J. H. & Pandis, S. N. Atmospheric chemistry and physics: from air pollution to climate change, 3rd edn, (Wiley, New York, USA, 2016).

[15] Wang Y (2014). Enhanced sulfate formation during China’s severe winter haze episode in January 2013 missing from current models. J. Geophys. Res. Atmos..

[16] Huang X (2014). Pathways of sulfate enhancement by natural and anthropogenic mineral aerosols in China. J. Geophys. Res. Atmos..

[17] Pandis SN, Seinfeld JH (1989). Mathematical modeling of acid deposition due to radiation fog. J. Geophys. Res. Atmos..

[18] Pye HOT (2020). The acidity of atmospheric aarticles and alouds. Atmos. Chem. Phys..

[19] Song S (2018). Fine particle pH for Beijing winter haze as inferred from different thermodynamic equilibrium models. Atmos. Chem. Phys..

[20] Guo H, Weber RJ, Nenes A (2017). High levels of ammonia do not raise fine particle pH sufficiently to yield nitrogen oxide-dominated sulfate production. Sci. Rep..

[21] Shi G (2017). pH of aerosols in a polluted atmosphere: source contributions to highly acidic aerosol. Environ. Sci. Technol..

[22] Shao J (2019). Heterogeneous sulfate aerosol formation mechanisms during wintertime Chinese haze events: air quality model assessment using observations of sulfate oxygen isotopes in Beijing. Atmos. Chem. Phys..

[23] Li J (2020). Stable sulfur isotopes revealed a major role of transition-metal-ion catalyzed SO_2_ oxidation in haze episodes. Environ. Sci. Technol..

[24] Jacob DJ (2000). Heterogeneous chemistry and tropospheric ozone. Atmos. Environ..

[25] Song S (2019). Possible heterogeneous chemistry of hydroxymethanesulfonate (HMS) in northern China winter haze. Atmos. Chem. Phys..

[26] Moch JM (2018). Contribution of hydroxymethane sulfonate to ambient particulate matter: a potential explanation for high particulate sulfur during severe winter haze in Beijing. Geophys. Res. Lett..

[27] Oblath SB, Markowitz SS, Novakov T, Chang SG (1982). Kinetics of the initial reaction of nitrite ion in bisulfite solutions. J. Phys. Chem..

[28] Martin LR, Damschen DE, Judeikis HS (1981). The reactions of nitrogen oxides with SO2 in aqueous aerosols. Atmos. Environ..

[29] Chang SG, Toossi R, Novakov T (1981). The importance of soot particles and nitrous acid in oxidizing SO_2_ in atmospheric aqueous droplets. Atmos. Environ..

[30] Jayne JT (2000). Development of an aerosol mass spectrometer for size and composition analysis of submicron particles. Aerosol Sci. Technol..

[31] Tian H (2015). Global methane and nitrous oxide emissions from terrestrial ecosystems due to multiple environmental changes. Ecosys. Heal. Sustain.

[32] Wallington TJ, Wiesen P (2014). N_2_O emissions from global transportation. Atmos. Environ..

[33] Park JY, Lee YN (1988). Solubility and decomposition kinetics of nitrous acid in aqueous solution. J. Phys. Chem..

[34] McKeen SA (1996). Hydrocarbon ratios during PEM-WEST A: a model perspective. J. Geophys. Res. Atmos..

[35] Brasseur, G. P. & Jacob, D. J. Modeling of atmospheric chemistry. (Cambridge University Press, 2017).

[36] Xu W (2019). NH_3_-promoted hydrolysis of NO_2_ induces explosive growth in HONO. Atmos. Chem. Phys..

[37] Pan Y (2016). Fossil fuel combustion-related emissions dominate atmospheric ammonia sources during severe haze episodes: evidence from ^15^N-stable isotope in size-resolved aerosol ammonium. Environ. Sci. Technol..

[38] Weber RJ, Guo H, Russell AG, Nenes A (2016). High aerosol acidity despite declining atmospheric sulfate concentrations over the past 15 years. Nat. Geosci..

[39] Wang G (2018). Particle acidity and sulfate production during severe haze events in China cannot be reliably inferred by assuming a mixture of inorganic salts. Atmos. Chem. Phys..

[40] Liu M (2017). Fine particle pH during severe haze episodes in northern China. Geophys. Res. Lett..

[41] Jacob DJ, Waldman JM, Munger JW, Hoffmann MR (1986). The H_2_SO_4_-HNO_3_-NH_3_ system at high humidities and in fogs: 2. Comparison of field data with thermodynamic calculations. J. Geophys. Res. Atmos..

[42] Shi G (2019). Aerosol pH dynamics during haze periods in an urban environment in China: use of detailed, hourly, speciated observations to study the role of ammonia availability and secondary aerosol formation and urban environment. J. Geophys. Res. Atmos..

[43] Xie Y (2015). Enhanced sulfate formation by nitrogen dioxide: Implications from in situ observations at the SORPES station. J. Geophys. Res. Atmos..

[44] Xue J, Yuan Z, Yu JZ, Lau AKH (2014). An observation-based model for secondary inorganic aerosols. Aerosol Air Qual. Res..

[45] Lee, Y. N., & Schwarts, S. E. Kinetic of oxidation of quueous sulfur(IV) by nitrogen dioxide. In precipitation Scavenging, Dry Deposition and Resuspension. **1** (eds Pruppacher, H. R. et al.), (Elsevier, New York, USA, 1983).

[46] Clifton CL, Altstein N, Huie RE (1988). Rate constant for the reaction of nitrogen dioxide with sulfur(IV) over the pH range 5.3–13. Environ. Sci. Technol..

[47] Jacob DJ (1985). Comment on “The photochemistry of a remote stratiform cloud” by William L. Chameides. J. Geophys. Res. Atmos..

[48] Liu Y (2020). The promotion effect of nitrous acid on aerosol formation in wintertime Beijing: possible contribution of traffic-related emission. Atmos. Chem. Phys. Discuss..

[49] Zhang W (2019). Variations and sources of nitrous acid (HONO) during a severe pollution episode in Beijing in winter 2016. Sci. Total. Environ..

[50] Zhang B, Zhang Y, Zhao X, Meng J (2018). Non-CO_2_ greenhouse gas emissions in China 2012: inventory and supply chain analysis. Earths Future.

[51] Zheng B (2018). Trends in China’s anthropogenic emissions since 2010 as the consequence of clean air actions. Atmos. Chem. Phys..

[52] Wang J (2019). Characterization of black carbon-containing fine particles in Beijing during wintertime. Atmos. Chem. Phys..

[53] Zhou W (2018). Production of N_2_O_5_ and ClNO_2_ in summer in urban Beijing, China. Atmos. Chem. Phys.

[54] Deng C, Yin X, Gan L (2014). Stratification characteristic analysis of atmospheric liquid water content and relative humidity during fog and haze weather in Beijing. Clim. Environ. Res..

[55] Quan J (2011). Analysis of the formation of fog and haze in North China Plain (NCP). Atmos. Chem. Phys..

[56] Yu Q (2019). Characteristics and secondary formation of water-soluble organic acids in PM_1_, PM_2.5_ and PM10 in Beijing during haze episodes. Sci. Total Environ..

[57] Turpin BJ, Lim H-J (2001). Species contributions to PM_2.5_ mass concentrations: revisiting common assumptions for estimating organic mass. Aerosol Sci. Technol..

