# Supplementary material for: Fast sulfate formation from oxidation of SO2 by NO2 and HONO observed in Beijing haze
Source: Nat Commun. 2020 Jun 5;11:2844. doi: 10.1038/s41467-020-16683-x (PMC7275061; doi:10.1038/s41467-020-16683-x)
Supplement: Supplementary file 1 — Supplementary Information [file 41467_2020_16683_MOESM1_ESM.pdf]

Supplementary Material for

Fast sulfate formation from oxidation of SO_2 by NO_2 and HONO observed in Beijing haze

Junfeng Wang[1, 2†], Jingyi Li[1†], Jianhuai Ye[2†], Jian Zhao[3], Yangzhou Wu[1,4], Jianlin Hu[1], Dantong Liu[4], Dongyang Nie[1,10], Fuzhen Shen[1], Xiangpeng Huang[1], Dan Dan Huang[5], Dongsheng Ji[3], Xu Sun[7], Weiqi Xu[3], Jianping Guo[8], Shaojie Song[2], Yiming Qin[2], Pengfei Liu[2], Jay R. Turner[9], Hyun Chul Lee[11], Sungwoo Hwang[11], Hong Liao[1],Scot T. Martin[2], Qi Zhang[6], Mindong Chen[1], Yele Sun[3], Xinlei Ge[1]*, and Daniel J. Jacob[2]*

[1]Harvard-NUIST Joint Laboratory for Air Quality and Climate, Jiangsu Key Laboratory of Atmospheric Environment Monitoring and Pollution Control, School of Environmental Science and Engineering, Nanjing University of Information Science and Technology, Nanjing 210044, China

[2]John A. Paulson School of Engineering and Applied Sciences, Harvard University, Cambridge, MA 02138

[3]State Key Laboratory of Atmospheric Boundary Layer Physics and Atmospheric Chemistry, Institute of Atmospheric Physics, Chinese Academy of Sciences, Beijing 100191, China

[4]Department of Atmospheric Sciences, School of Earth Sciences, Zhejiang University, Hangzhou 310007, China

[5]State Environmental Protection Key Laboratory of Formation and Prevention of Urban Air Pollution Complex, Shanghai Academy of Environmental Sciences, Shanghai 200233, China

[6]Department of Environmental Toxicology, University of California Davis, Davis, CA 95616, USA

[7]State Key Laboratory of Urban and Regional Ecology Research Center for Eco-Environmental Sciences, Chinese Academy of Sciences, Beijing 100085, China

[8]State Key Laboratory of Severe Weather, Chinese Academy of Meteorological Sciences, Beijing 100081, China

[9]Department of Energy, Environmental and Chemical Engineering, Washington University in Saint Louis, St. Louis, MO 63130, USA

[10]School of Atmospheric Sciences, Nanjing University, Nanjing 210023, China

[11]Samsung Advanced Institute of Technology, Suwon-si, Gyeonggi-do, 16678, Republic of Korea

[†]These authors contributed equally to the work.

*Corresponding authors: Xinlei Ge (Email: caxinra@163.com); Daniel J. Jacob (Email: djacob@fas.harvard.edu)

Supplementary Table 1: Henry's law and acid dissociation constants[a]

Equilibrium	Equilibrium constant at 271 K
$H_2O \leftrightarrow H^+ + OH^-$	1.0×10^{-15} M
$CO_2(g) + H_2O \leftrightarrow CO_2 \cdot H_2O$	7.7×10^{-2} M atm^{-1}
$CO_2 \cdot H_2O \leftrightarrow H^+ + HCO_3^-$	5.9×10^{-7} M
$HCO_3^- \leftrightarrow H^+ + CO_3^{2-}$	8.5×10^{-11} M
$SO_2(g) + H_2O \leftrightarrow SO_2 \cdot H_2O$	3.3 M atm^{-1}
$SO_2 \cdot H_2O \leftrightarrow H^+ + HSO_3^-$	2.5×10^{-2} M
$HSO_3^- \leftrightarrow H^+ + SO_3^{2-}$	1.1×10^{-7} M
$NH_3(g) + H_2O \leftrightarrow NH_3 \cdot H_2O$	2.5×10^{2} M atm^{-1}
$NH_3 \cdot H_2O \leftrightarrow NH_4^+ + OH^-$	4.0×10^{-5} M
$NO_2(g) + H_2O \leftrightarrow NO_2 \cdot H_2O$	2.3×10^{-2} M atm^{-1}
$HONO(g) \leftrightarrow HONO \cdot H_2O$	2.4×10^{2} M atm^{-1}
$HONO \cdot H_2O \leftrightarrow NO_2^- + H^+$	3.4×10^{-4} M

[a] Equilibrium constants as given by Seinfeld and Pandis[1] and adjusted to 271 K using the van't Hoff equation.

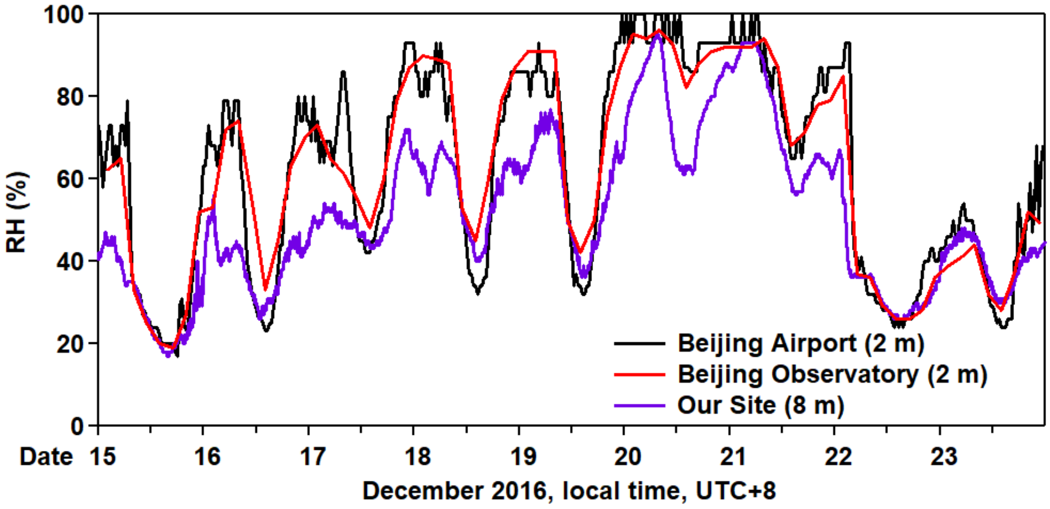

Supplementary Figure 1: Relative humidity during the haze event. Observed relative humidity (RH) at Beijing International Airport, Beijing Observatory Meteorological Station, and our sampling site during the December 16-22, 2016 haze event. UTC+8 refers to Coordinated Universal Time + 8 hours and indicates the local solar time in hours.

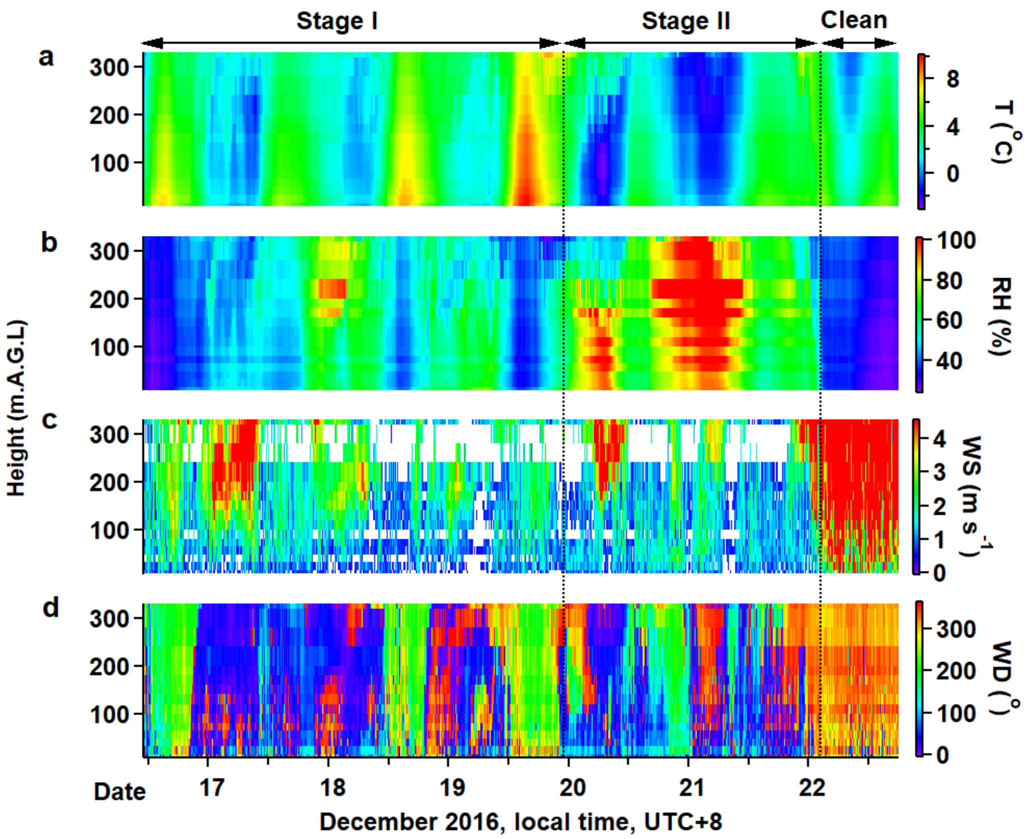

Supplementary Figure 2: Meteorological profiles above the sampling site. Vertical profiles above ground level (AGL) of (a) temperature (T), (b) relative humidity (RH), (c) wind speed (WS) and (d) wind direction (WD) measured at the IAP 325-m meteorological tower during the December 16-22, 2016 haze event.

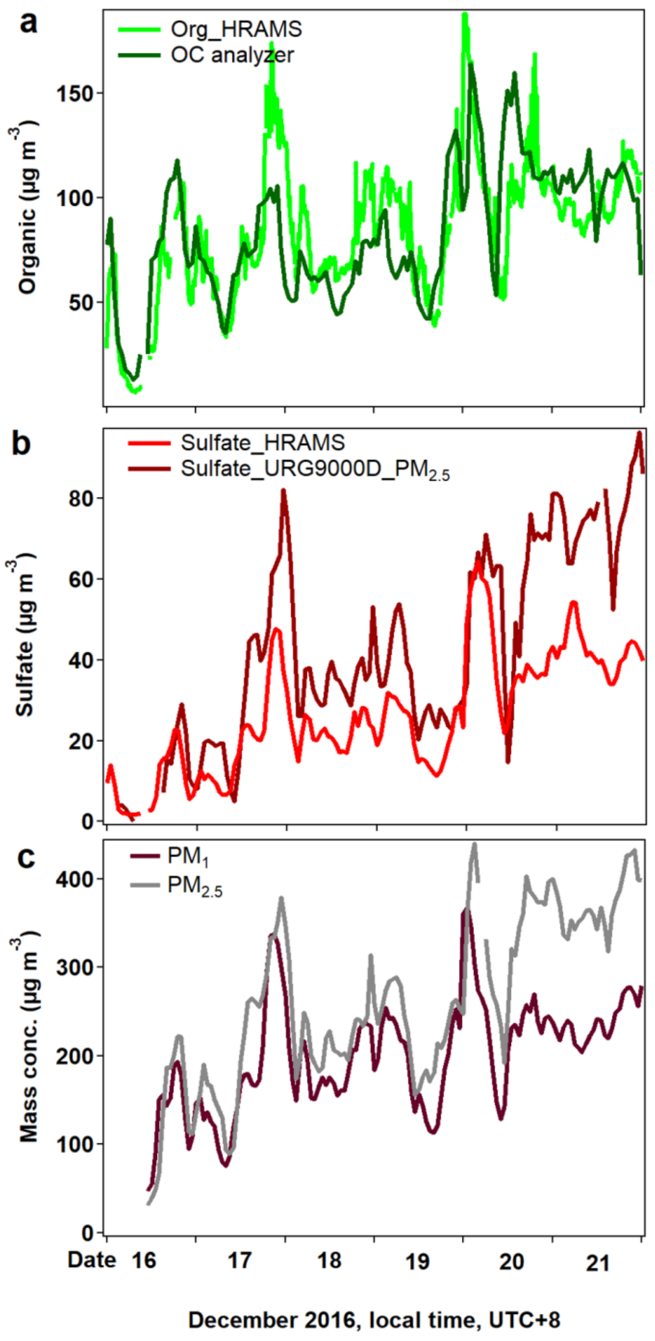

Supplementary Figure 3: Differences between PM_1 and $PM_{2.5}$. The plots compare PM_1 and $PM_{2.5}$ (a) organic, (b)sulfate and (c) total mass concentrations during the December 16-22, 2016 haze event. PM_1 organic and sulfate mass concentrations were measured by HR-AMS. $PM_{2.5}$ sulfate mass concentrations were measured with a URG-9000D Ambient Ion Monitor. $PM_{2.5}$ organic carbon (OC) mass concentrations were measured by a thermo-optical ECOC analyzer (Sunset Laboratory Inc., Tigard, Oregon). We assumed an organic mass to organic carbon ratio of 1.9[2] to convert the OC measurement to total organic mass. PM_1 is the total particulate mass concentration inferred from the HR-AMS data. $PM_{2.5}$ was measured 4 km to the northeast of the IAP site.

Supplementary References

1 Seinfeld, J. & Pandis, S. in *Atmospheric Chemistry and Physics: From Air Pollution to Climate Change, 3rd Edition* Ch. 26, 1152 (2016).

2 Turpin, B. J. & Lim, H.-J. Species Contributions to PM2.5 Mass Concentrations: Revisiting Common Assumptions for Estimating Organic Mass. *Aerosol Sci Tech* **35**, 602-610, doi:10.1080/02786820119445 (2001).
